# Heart rate variability alterations in takotsubo syndrome and related association with psychological factors: a systematic review and meta-analysis

**DOI:** 10.1038/s41598-023-47982-0

**Published:** 2023-11-25

**Authors:** Gianluca Cruciani, Marco Cavicchioli, Gaetano Tanzilli, Annalisa Tanzilli, Vittorio Lingiardi, Federica Galli

**Affiliations:** 1 Department of Dynamic and Clinical Psychology, and Health Studies, Sapienza University of Rome, Via degli Apuli 1, 00185 Rome, Italy; 2 Department of Psychology, University “Vita-Salute San Raffaele”, Via Stamira d’Ancona 20, Milan, Italy; 3 Department of Clinical Internal, Anesthesiologic and Cardiovascular Sciences, Sapienza University of Rome, Viale del Policlinico 155, Rome, Italy

**Keywords:** Cardiology, Health care

## Abstract

Psychological factors may have a precipitant role in takotsubo syndrome (TS). Aberrant Heart Rate Variability (HRV) has been reported in TS, suggesting inflexibility of the autonomous nervous system. Nevertheless, results on HRV alterations and their link with psychological factors in TS are conflicting. This work aimed to systematically explore whether TS may be associated with HRV alterations and their association with specific psychological profiles in TS patients. A literature search was conducted across databases (Pubmed, Scopus, PsycInfo, Web of Science) and empirical studies including TS patients which were evaluated in one or more HRV indices were retrieved. HRV and psychological outcomes were extracted. 10 empirical studies with 194 TS patients were included. Results showed significant alteration of HRV in TS patients, with indices compared to controls, and a progressive increase over time. Nevertheless, retrieved data presented mixed results, as also shown by a large heterogeneity in the meta-analytic findings. 2 studies found significant relationships between HRV alterations and trait—rather than state—psychological outcomes (i.e., coping strategies and emotional arousal), pointing to the need to explore the role of psychological vulnerabilities, rather than single traumatic stressors, in the association between HRV and TS.

## Introduction

Takotsubo syndrome (TS), also known as broken heart syndrome, apical ballooning syndrome, or stress cardiomyopathy, is a coronary syndrome characterized by a transient heart failure that mimics acute myocardial infarction. TS, occurring after emotional stressors that cause microvascular spasm with temporary myocardial stunning, is generally associated to good prognosis with full recovery of left ventricular contractility^1^. Differently, direct myocardial injury with activation of cell survival cascade, as observed when a physical trigger is present, may determine severe and persistent LV dysfunction that result in a worse long-term prognosis^2^. This suggest that different structural concomitant conditions may play a main role in determining its unfavorable outcome^3^.

On the other hand, the aetiology of TS is not yet clear, and several complex pathophysiological mechanisms have been hypothesized to be responsible for TS^4,5^. It has been observed that around 1–3% of patients presenting with acute coronary syndrome may be diagnosed as TS patients, with the prevalence being even higher – up to 5–6%—in the female population, especially among postmenopausal women^6,7^. Notably, 80% of TS patients report a precipitant major stressful event prior to the acute cardiac event, suggesting a pivotal role of psychological factors in this syndrome^8^. A recent systematic review^9^ analysed the role of psychological factors in exacerbating and maintaining TS. On the one hand, concurrent stressful, traumatic life-events, either emotional or physical factors have often been identified as triggers or antecedents of TS^10–12^. On the other hand, anxiety^13,14^, depression^15,16^ and maladaptive personality factors appeared to be stable and homogeneous characteristics that differentiate TS patients from healthy controls^17,18^.

From a psychophysiological standpoint, heart rate variability (HRV) — a measure of the variability of adjacent heart beats which reflects the interplay between the parasympathetic and the sympathetic nervous systems—has often been employed as an index of effective emotion-regulation and flexible adaptation to environmental demands^19,20^. HRV can be analysed in the time domain, in the frequency domain, and with non-linear indices that consider the complex and erratic fluctuations on the autonomic nervous system^21–23^ (a synthesis of the main HRV parameters is provided in Table S1 in supplementary material).

Interestingly, weaker sympathovagal balance—indicated by aberrant HRV—has been reported both during the acute phase of TS^24^ and in the subsequent interval^25^, pointing to an inflexibility of the autonomous nervous system, which has been linked to poor health outcomes^26^. Nevertheless, systematic results on HRV alterations and their possible link with psychological factors in TS patients are sparse, and often derived from case-reports^27,28^. For these reasons, the principal aim of the current systematic review and meta-analysis is to explore whether TS may be associated with specific autonomic alterations in the HRV domain. Furthermore, given that HRV has been often employed as a marker of impaired abilities of emotion regulation and environmental adaptation, a second aim of this work is to assess whether possible autonomic alterations may be associated with specific psychological profiles in TS patients. Consistently, empirical human studies on TS patients which also underwent autonomic assessment were retrieved and reviewed according to specific predefined criteria, and data on HRV and its relationship with psychological features have been qualitatively and quantitatively analysed.

## Results

202 potentially eligible manuscripts were retrieved from the initial databases search; after duplicates removal, 151 articles were first screened by reading title and abstract. 95 manuscripts survived the first step and underwent further screening by applying inclusion and exclusion criteria: of these, 10 met all the predefined criteria and were thus included in the present systematic review. Table 1 displays an overview of the general characteristics of the included studies. Tables S2 and S3 in supplementary material provide further information about the quality assessment of the articles.

**Table 1:** Overview of the characteristics of the studies included in the systematic review.

Study	TS: N, (mean age ± SD); N females (f)	Controls: N, (mean age ± SD); N females (f)	Controls’ diagnosis	Assessment (time)	HRV outcome measures	Psychological outcome measures	Significant findings	NOS score
Akashi et al., 2007*^42^	10 (70.1 ± 13.7); 8 f.	10 (70.0 ± 6.1); not defined	HV	2 ± 1 days after admission (0 months) and 3.0 ± 0.2 months after discharge (3 months)	Mean RR; SDNN; SDANN; pNN50; RMSSD; LF; HF; TF; LF/HF	None	SDNN: TS 0 months < HV (*p* = 0.007); TS 0 months < TS 3 months (*p* = 0.01) SDANN: TS 0 months < HV (*p* = 0.01); TS 0 months < TS 3 months (*p* = 0.03) TF: TS 0 months < HV (*p* = 0.005) LF: TS 0 months < HV (*p* = 0.01); TS 3 months < HV (*p* = 0.02) LF/HF: TS 3 months < HV (*p* = 0.006)	9
Bonnemeier et al., 2010**^45^	27 (68.6 ± 11); 26 f.	10 (64.5 ± 8); 10 f.	MB	Third day after hospital admission	Mean RR; SDNN; SDANN; RMSSD; LF; HF; LF/HF; 1/f PLS; ApEn; AC; DC	None	SDANN: TS > MB (*p* = 0.029) LF: TS < MB (*p* = 0.028) HF: TS > MB (*p* = 0.034) LF/HF: TS < MB (*p* = 0.031) 1/f PLS: TS < MB (*p* = 0.008) ApEn: TS > MB (*p* = 0.009) AC: TS < MB (*p* = 0.005) DC: TS > MB (*p* = 0.004)	7
Collste et al., 2014*^48^	22 (63.2); 21 f.	22 (63.6); 21 f.	HV	6 months after the acute event	SDNN; SDANN	Mental Stress Test	SDNN pre-stress Δ: TS > HV (*p* = 0.002)	7
Krstacic et al., 2012*^41^	25 (60.2 ± 8.3); 25 f.	50 (63.1 ± 7.2); 50 f.	HV	T1: hospital admission T2: after few days when patients have already received beta blockers treatment T3: at hospital discharge	Mean RR; SDNN; SDANN; LF; HF; LF/HF; H; DFA α1; DFA α2; ApEn	None	Mean RR: TS-T1 < HV (*p* < 0.05) SDNN: TS-T1 < HV (*p* < 0.05); TS-T2 < TS-T3 (*p* < 0.05) SDANN: TS-T1 < HV (*p* < 0.05); TS-T2 < TS-T3 (*p* < 0.05) LF: TS-T1 > HV (*p* < 0.05); TS-T2 > TS-T3 (*p* < 0.05) HF: TS-T1 < HV (*p* < 0.05); TS-T2 < TS-T3 (*p* < 0.05) LF/HF: TS-T1 > HV (*p* < 0.01); TS-T2 > TS-T3 (*p* < 0.01) H: TS-T1 < HV (*p* < 0.001); TS-T2 < TS-T3(*p* < 0.001) DFA α1: TS-T1 > HV (*p* < 0.001); TS-T2 > TS-T3 (*p* < 0.001) DFA α2: TS-T1 > HV (*p* < 0.05); TS-T2 > TS-T3 (*p* < 0.01) ApEn: TS-T1 < HV (*p* < 0.001); TS-T2 < TS-T3 (*p* < 0.001)	8
Lazzeroni et al., 2022*^46^	10 (69 ± 7); 9 f.	9 (72 ± 7); 8 f.	HV	12–24 months following the acute TS episode	SDNN; pNN50; RMSSD; VLF; LF; HF; LF/HF; SamEn; FD	16PF; AAQ-II; STAI; BDI-II; COPE	SDNN: TS < HV (*p* = 0.048) pNN50: TS < HV (*p* = 0.003) RMSSD: TS < HV (*p* = 0.015) HF: TS < HV (*p* = 0.030) LF/HF: TS > HV (*p* = 0.012) FD: TS > HV (*p* = 0.013)	8
Mayer et al., 2016**^50^	26 (64.9 ± 13.5); 26 f.	Normative data	–	Median time from the TS event to the time of investigation: 17.5 months	SDNN; SDANN; RMSSD	HADS; ESS; FSS; TICS; FPI-R; MOCA; Five point test; Stroop Task	Only descriptive data	2
Norcliffe-Kaufmann et al., 2016*^47^	10 (61.7 ± 11.7); 10 f.	10 (age not reported); 10 f.	HV	Mean time from the TS event to the time of investigation: 42.1 months	SDNN; pNN50; RMSSD; LF; HF	STAI; Somatic vigilance scale; Stroop Task; Event recall	SDNN: TS < HV (*p* = 0.035) pNN50: TS < HV (*p* = 0.014) RMSSD: TS < HV (*p* = 0.031) HF: TS < HV (*p* = 0.079)	7
Ortak et al., 2009**^43^	39 (67.8 ± 10.5); 38 f.	Same group at different times	–	T1: day 1 after admission T2: day 2 after admission T3: day 3 after admission T4: 3 months after hospitalization	Mean RR; SDNN; SDNNi; SDANN; RMSSD; TI	None	SDNN: TS-T1 < TS-T3 (*p* = 0.041); TS-T1 < TS-T4 (*p* = 0.002) SDNNi: TS-T1 < TS-T3 (*p* = 0.050); TS-T1 < TS-T4 (*p* = 0.003) SDANN: TS-T1 < TS-T3 (*p* = 0.005); TS-T1 < TS-T4 (*p* < 0.001) RMSSD: TS-T1 < TS-T3 (*p* = 0.048); TS-T1 < TS-T4 (*p* = 0.050) TI: TS-T1 < TS-T4 (*p* = 0.020)	3
Waldenborg et al., 2011**^44^	13 (70); 13 f.	Same group at different times	–	T1: within 3 days following symptom presentation T2: after 3 months	Mean RR; SDNN; SDANN; TINN; pNN50; SDSD; RMSSD; VLF; LF; HF; TF	PTSS-10; MADRS-S	SDNN: TS-T1 < TS-T2 (*p* = 0.008) SDANN: TS-T1 < TS-T2 (*p* = 0.008)	3
Watson et al., 2022***^49^	12 (67.3 ± 2.9); 12 f.	12 (66.2 ± 2.5); 12 f.	CP	Three registrations during experimental task in a follow-up visit (no time specified) T1: baseline condition T2: hyperventilation condition T3: 5 min after	SDNN	Hyperventilation induced anxiety	SDNN: increased in response to hyperventilation (*p* < 0.001) but no differences between cases and controls	7

* = Case–control study; ** = Prospective study; *** = Randomised cross-over trial; 16PF, 16 personality factors – C form; AAQ-II, acceptance and action questionnaire-II; AC, acceleration capacity; ApEn, approximate entropy; BDI-II, Beck depression inventory-II; COPE, coping orientations to problems experienced; CP, Patients seen by the cardiology service for investigation of chest pain or palpitations but free of significant cardiac pathology; DC, deceleration capacity; DFA, detrended fluctuation analysis; ESS, Epworth sleepiness scale; FD, fractal dimension; FPI, Freiburg personality inventory-R; FSS, fatigue severity scale; H, Hurst exponent; HADS, hospital anxiety and depression scale; HF, high frequency; HV, healthy volunteers; LF, low frequency; LF/HF, ratio between LF and HF; MADRS-S, Montgomery-Åsberg depression rating scale, self rated version; MB, midventricular ballooning; MOCA, Montreal cognitive assessment; PLS, power law slope; pNN50 = percentage differences between adjacent normal RR intervals exceeding 50 ms; PTSS-10, post-traumatic stress syndrome 10-questions inventory; RMSSD, root-mean-square successive difference between adjacent normal NN intervals; RR, time duration between two consecutive R waves of the ECG; SDANN, standard deviation of the average normal RR intervals for every 5 min of ECG recording; SDNN, standard deviation of normal RR intervals in ECG recording; SDNNi, mean standard deviation of RR intervals for all 5 min segments; SDSD, mean of standard deviations of normal RR intervals for all 5-min segments; STAI, , state-trait anxiety inventory; TF, total frequency; TI, triangular index; TICS, Trier inventory for chronic stress; TINN, baseline width of the minimum square difference triangular interpolation of the highest peak of the histogram of NN intervals; VLF, very low frequency.

### General characteristics of the studies

Overall TS sample sizes varied between n = 10 and n = 39. Globally, a total of n = 194 TS patients were evaluated. 96.91% of the global TS sample was composed of female patients, with 5 studies including only female patients in their sample. Mean age of TS samples across the studies ranged from 60.2 ± 8.3 and 70.1 ± 13.7 years. Looking at control groups, sample sizes varied between 9 and 50 participants, for a total of n = 123 controls recruited. Control groups were age- and sex-matched in most of the retrieved studies, although in one article the sex of control participants was not reported. Mean age of control samples ranged from 63.1 ± 7.2 to 72 ± 7 years, although in one study the controls mean age is missing. Control groups were composed of healthy volunteers in 5 studies, patients with midventricular ballooning in 1 study, and patients seen by the cardiology service for investigation of chest pain or palpitations but free of significant cardiac pathology in 1 other study. With respect to the remaining studies, in one case TS group performances were compared with normative data, whereas in other two studies outcomes were recorded on the same TS sample but at different times. Quality indices of the retrieved studies varied between 2 and 7 points, with a median score of 5.5.

### TS and HRV indices

Retrieved articles result heterogeneous in terms of HRV indices employed. The median value of the number of HRV indices included in each study is 7.5, with a range between 1 and 11. The most used HRV indices belong to the time domain, with a total of 10 articles evaluating at least one time domain index. Of these, 4 studies only considered HRV indices belonging to the time domain, 3 included HRV indices in both the time domain and the frequency domain, and 3 used both time domain and frequency domain indices as well as non-linear analyses. The most used index is SDNN, which was considered in all the 10 articles, followed by SDANN and RMSSD (7 articles), LF and HF (6 articles), mean RR (5 articles), LF/HF and pNN50 (4 articles), VLF, TF and ApEn (2 articles); all the remaining indices (i.e., SDNNi, SDSD, TI, TINN, 1/f PLS, AC, DC, DFA α1, DFA α2, FD, H, SamEn) were only considered once.

#### Time domain

Considering mean RR, only 1 study found significant lower values in TS patients compared to healthy controls at hospital admission, but no differences were found after few days from the acute event or at hospital discharge^29^. Other studies that evaluated mean RR found no differences between TS patients and healthy controls at hospital admission or 3 months after discharge^30^, no improvement of mean RR in the 3 months following the acute event^31,32^, and no significant differences between TS patients and patients with midventricular ballooning^33^.

Regarding SDNN, 4 studies compared TS patients with healthy controls and found significant lower levels of SDNN in TS patients^29,30,34,35^; only 1 study found higher SDNN levels in TS patients than healthy controls, although HRV was evaluated 6 months after the acute event^36^. Two other studies evaluated SDNN levels of TS patients at different times after the acute event, showing a progressive increase of SDNN as a function of time^31,32^: these data are in line with results by Krstacic and colleagues^29^ and Akashi and colleagues^30^ showing no differences in terms of SDNN between healthy controls and TS patients at hospital discharge and 3 months after discharge respectively, suggesting a potential spontaneous recover of SDNN after the acute event. When TS group was compared to patients with midventricular ballooning^33^ and patients admitted to cardiology service for an investigation of chest pain or palpitations^37^, no significant differences were found considering the SDNN index. In one case^38^, TS patients’ SDNN was compared with normative data and values were within the expected limits.

Looking at the SDANN, 2 studies reported lower values for TS patients compared to healthy controls when evaluated right after the acute event^29,30^. Nevertheless, no differences emerged in studies which evaluated SDANN levels in TS patients and healthy controls at hospital discharge^29^, 3 months^30^ or 6 months after the acute event^36^. Similarly, 2 other studies showed an increase of SDANN levels after 3 months after the acute event^31,32^, suggesting a potential spontaneous recover of this index, as observed for SDNN. One study showed that TS patients displayed significant higher values of SDANN compared to patients with midventricular ballooning^33^. On the contrary, TS patients’ SDANN values were within the expected limits referring to normative data^38^.

Concerning pNN50, Akashi and colleagues^30^ found no significant differences between TS patients and healthy controls neither right after the acute event nor 3 months after hospital discharge; similar findings were found by Waldenborg and colleagues^32^ which showed no differences in TS patients’ pNN50 over 3 months after the cardiac event. On the contrary, 2 studies found significant lower levels of pNN50 in TS patients compared to healthy controls considering a long follow-up period after the acute event, namely 12–24 months^34^ and 42 months^35^.

With respect to RMSSD, results are mixed. In 2 studies, TS patients reported significant lower level of RMSSD than healthy participants even after 12–24 months^34^ or 42 months^35^ after the acute event, whereas 2 other studies showed no differences between TS patients and healthy participants neither during nor after 3 months from the acute event^30^. Similarly, Waldenborg and colleagues^32^ reported no differences in terms of RMSSD between the moment of the acute event and 3 months later; whereas, Ortak and colleagues^31^ showed increased levels of this index during a 3-month follow-up period after hospitalization. One study^33^ highlighted no significant differences between TS patients and ones with midventricular ballooning in terms of RMSSD. Similarly, no significant differences were found compared the TS patients’ RMSSD to normative data^38^, although patients with remaining symptoms after their acute event showed significantly higher values compared to patients without residual symptoms. In one study^38^, TS patients’ RMSSD was compared with normative data and values were within the expected limits, although patients with remaining symptoms after their acute event showed significantly higher values compared to patients without residual symptoms.

Lastly, a significant increase of SDNNi and TI was observed in TS patients in the 3 months following the acute event^31^. On the contrary, SDSD and TINN remained unchanged considering the same period^32^.

#### Frequency domain

Looking at LF, 1 study showed lower level of this index for TS patients compared to healthy controls both right after and 3 months after the acute event^30^. On the contrary, 2 studies reported no significant differences between TS patients and healthy controls after 12–24 months^34^ or 42 months^35^. Waldenborg and colleagues^32^ showed no increases in LF among TS patients between the time of symptom presentations and after 3 months from the acute event. One study found higher LF levels in TS patients at hospital admission than healthy controls^29^. Moreover, TS patients showed lower values of LF were compared to patients with midventricular ballooning^33^.

Referring to HF, 3 studies found lower level of HF in TS patients compared to healthy controls at hospital admission^29^, and at 12–24 months^34^ and 42 months^35^ after the acute event. Akashi and colleagues^30^ reported no differences between TS patients and healthy controls neither at hospitalization nor 3 months after the acute event. No changes in HF were observed by Waldenborg and colleagues^32^ in TS patients from hospitalization to 3 months after discharge. In addition, TS patients showed significantly higher HF levels than midventricular ballooning^33^.

Referring to LF/HF, 1 study found no differences between TS patients and healthy controls right after the acute phase but showed a significant decrease of TS patients’ LF/HF index in the following 3 months when compared to controls^30^. Krstacic and colleagues^29^ found higher LF/HF values for TS patients compared to healthy controls at hospital admission time and a decrease of LF/HF in the following days until hospital discharge, whereas Lazzeroni and colleagues^34^ showed higher LF/HF values among TS patients than in healthy controls also 12–24 months after the acute event. Ultimately, TS patients were characterized by significant lower values of LF/HF than patients with midventricular ballooning^33^.

No differences in VLF values were found between TS patients and healthy controls evaluated after 12–24 months from the acute event^34^, nor changes over the 3 months following the cardiac event were observed^32^. No differences in the TF index were found between TS patients and healthy controls at hospital admission or 3 months after discharge^30^, nor changes over the 3 months following the cardiac event were observed^32^.

#### Non-linear indices

Lower levels of H were observed in TS patients compared to healthy controls at hospital admission, with a significant increase of H values from hospital admission to hospital discharge, whereas the opposite pattern was found for DFA α1 and DFA α2, with higher level in TS patients than healthy controls at hospital admission and a significant decrease from hospital admission to hospital discharge^29^. Lower ApEn values were observed in TS patients compared to healthy controls at hospital admission, with a significant increase of ApEn index from admission to hospital discharge^29^ whereas higher levels of ApEn were found in TS patients when compared to patients with midventricular ballooning^33^. Comparing the clinical and healthy control groups 12–24 months after the acute episode, higher levels of FD were found among TS patients whereas no significant differences were observed for SamEn^34^. Significant lower level of 1/f PLS and AC, but higher level of DC, were found in TS patients compared with patients with midventricular ballooning^33^.

### Relation between HRV and psychological profile in TS patients

Only 6 of the 10 retrieved articles took into consideration psychological factors, of which 3 evaluated HRV while performing tasks whereas 3 assessed HRV at rest.

Concerning task-dependent HRV, Collste and colleagues^36^ administered the Mental Stress Test to a cohort of 22 TS patients compared with 22 age- and sex-matched healthy controls 6 months after the acute event. They found no group difference in terms of SDNN and SDANN indices neither at baseline nor during the Mental Stress Test. Norcliffe-Kaufmann and colleagues^35^ showed that anxiety and somatic vigilance scores were similar across TS and healthy participants groups. The study did not report correlation between HRV parameters with cognitive and emotional tests., Nevertheless, authors highlighted a similar increase in heart rate and circulating catecholamines in response to the cognitive Stroop test in both groups. But a more variable systolic blood pressure in TS patients than in healthy controls. Interestingly, the emotional arousal evoked by recalling the triggering event led to a higher increase in blood pressure than cognitive arousal among TS patients. Watson and colleagues^37^ showed that HRV in terms of SDNN increased in response to a stress intervention compared to diaphragmatic breathing, but no differences were observed between TS patients and healthy controls. Furthermore, perceived anxiety measured through a self-report instrument after the stress condition did not differ between groups.

Regarding articles that evaluated HRV at rest conditions, Lazzeroni and colleagues^34^ showed that TS patients had significantly higher scores on trait anxiety, tension personality factor, and transcendental orientation coping mechanisms than healthy controls. Interestingly, a negative correlation between LF/HF and coping orientation was found, suggesting a possible association between autonomic impairment and coping mechanisms adopted even long after the acute event. Mayer and colleagues^38^ screened 26 patients with TS for psychiatric comorbidities, chronic stress, and personality traits. They showed that 57.7% of e patients had psychiatric comorbidities: 18/26 suffered from depressive mood, 1/26 suffered from phobia, 2/26 reported lifetime a panic attack, 2/26 had a history of anorexia nervosa, 1/26 attempted suicide and 2/26 reported physical abuse. TS patients also displayed high self-report levels of anxiety (i.e., Hospital Anxiety and Depression Scale) and chronic stress (i.e., Trier Inventory for Chronic Stress). Nevertheless, authors found no significant correlations between HRV parameters and scores in either subscale of the questionnaires. Similarly, Waldenborg and colleagues^32^ reported no statistically significant association between HRV indices with post-traumatic stress (i.e., Post-traumatic Stress Syndrome 10-Questions Inventory) and depressive symptoms (i.e., Montgomery-Åsberg depression rating scale). However, one patient fulfilled criteria for posttraumatic stress syndrome and 4 had borderline scores at 3 months follow-up.

### Meta-analytic findings

Five studies^29,30,33,34,36^ (N = 195 subjects; TS: N = 94) reported data that allowed to estimate 41 ESs used to conduct meta-analytic procedures. Specifically, data analyses were based on HRV indices measured at resting-state after the acute episode of patients with TS compared to HCs. With respect to an overall alteration of HRV, the 3-level model showed large and significant differences between patients with TS and HCs (*d*_*w*_ = *0.9*1 [0.65–1.29]; *p* < 0.001)*.* The 2-level model (*d*_*w*_ = *0.9*7 [0.66–1.29]; *p* < 0.001) was a more parsimonious solution that adequately fitted to data (χ_(1)_ = 0.96; ns). The analysis found a large heterogeneity of ESs (*I*^2^ = 85.67%). The meta-regression did not reveal a significant impact of variables (i.e., year of publication, sample size, age, sex, length of HRV record, time of HRV assessment after the acute episode) included in the statistical model, with exception of specific HRV (*F*_(19, 21)_ = 2.54; *p* < 0.05). Particularly, the non-linear index H (*d*_*w*_ = *3.95* [1.89–6.00]; *p* < 0.001) and DFA α1 (*d*_*w*_ = *3.74* [1.07–5.77]; *p* < 0.001) highlighted very large and significant pooled ESs, as well as the LF index (*d*_*w*_ = *1.86* [0.22–3.50]; *p* < 0.05) within the frequency domain. On the contrary, the other HRV indices showed no significant pooled ESs. The Egger’s regression did not find bias of publication (a detailed description of meta-analytic results is provided in Table S4 in supplementary material).

## Discussion

Results from both the systematic review and the meta-analysis are consistent in showing significant alteration of HRV in patients suffering from TS. In general, we found that HRV indices from both the time and the frequency domain, as well as non-linear indices, appear to be lower in TS patients compared to controls, with a progressive increase over time. Nevertheless, retrieved data presented mixed results, as also shown by a large heterogeneity across studies in the meta-analytic findings, pointing to the need for a deeper assessment of HRV course over time in TS patients.

Both time-domain and frequency-domain HRV indices analysed in the present work support the perspective of a sustained autonomic imbalance between sympathetic and parasympathetic systems in TS. For instance, SDNN and SDANN indices have been consistently indicated to be acute phase predictors of scarce prognoses in TS across the retrieved studies, mirroring a relative sympathetic predominance^39^. Although being low during the acute phase, SDNN and SDANN have been shown to recover over time after the cardiac event, suggesting a spontaneous normalization possibly mediated by several, unclear mechanisms, including a modulation of the sinus node and the vagal tone or changes in the renin-angiotensin activities, in the chemoreceptor function or in the respiratory pattern^30^. Nevertheless, studies also showed that RMSSD index was altered (either significantly higher or lower) in TS patients long after the acute event. On the one hand this suggests that parasympathetic activity may not spontaneously recover in this syndrome. On the other hand, it could be a useful indication for clinicians assessing autonomic function in TS, which should consider HRV indices differentially as a function of time elapsed from the acute event.

Concerning HRV non-linear indices, loss of regularity and predictability of the time series may reflect a reduced complexity of heart rate dynamics similar to what observed in patients with postoperative cardiac surgery complications^40^ or in other ischaemic or dilatative cardiomyopathies^41^. Altered cardiovascular regulation identified by non-linear indices of HRV have been thus proposed as a sign of an underlying structural – and not only functional—cardiovascular disease and may carry prognostic information^29^.

The significance of HRV assessment in several cardiovascular diseases, such as congestive heart failure, acute myocardial infarction, cardiac arrest, and supraventricular and ventricular arrhythmias, has been acknowledged since 1996 in published guidelines addressing HRV measurement standards^42^, and HRV has been shown to predict cardiovascular event in healthy populations, as well as the risk of rehospitalization and death in patients with cardiovascular diseases. For instance, a meta-analysis by Hillebrand and colleagues^43^ conducted on populations without known cardiovascular disease showed that impaired HRV values were positively associated with a 32–45% increased risk of a first cardiac event. A recent systematic review^44^ on patients with ST-elevation myocardial infarction (STEMI) demonstrated that lower time-domain HRV parameters and a higher LF/HF ratio were associated with higher mortality during follow-up, even in patients treated mainly with percutaneous coronary interventions. Similarly, also nonlinear HRV indices have been shown to be significant predictors of adverse outcomes (i.e., rehospitalization and death) in acute coronary syndrome^45^. Further evaluations are needed to assess whether HRV could have a predictor role of rehospitalization and mortality also in TS patients, considering that survivors of TS have been shown to have significantly higher associated mortality rates than survivors of STEMI^58^ as well as frequent hospital readmission within 1–3 months from discharge^47–49^.

In fact, taken together, results from the present work describe an autonomic phenotype of TS patients characterized by an enhanced sympathetic activation and a reduced parasympathetic modulation of the heart. Such characteristics, in particular lower parasympathetic inhibition capacities, have been often associated with myocardial infarction and heart failure^50,51^. Moreover, scientific literature reported cases of TS following parasympathetic denervation during cardiac ablation surgery, which may predispose to TS by causing an exaggerated sympathetic activity increase and labile hypertension^52,53^. Sympathetic disinhibition via a reduction of the parasympathetic control is typical in stressful situations in which a psychological event triggers the “fight or flight” response^54^. Specifically, heart rate, blood pressure and cardiac output are enhanced by adrenergic neurohumoral activation and prepare individuals for facing situational stressors. Although these features are considered short-term beneficial coping strategies in many acute situations, a long-term activation of the sympathetic system may affect the integrity of the cardiovascular system and, it is considered a risk factor for heart diseases^55^. Notably, precipitating psychological triggers are often reported in TS, with 80% of TS patients presenting a major stressful event prior to the acute cardiac event, suggesting a pivotal role of psychological factors in this syndrome^8^.

For these reasons, a second aim of this work was to assess whether possible autonomic alterations may be associated with specific psychological profiles in TS patients. Only 2 of the 6 retrieved studies that assessed possible association between HRV and psychological features in TS patients found significant relationships. Particularly, Lazzeroni and colleagues^34^ reported a negative correlation between LF/HF and transcendental orientation coping, suggesting a possible association between autonomic impairment and coping mechanisms adopted even long after the acute event; Norcliffe-Kaumann and colleagues^35^ showed that the emotional arousal evoked by recalling a stressful event led to a higher increase in blood pressure among TS patients compared to controls. Notably, all the other studies failed in demonstrating associations between HRV aberrations and psychological variables in TS patients. Such inconsistencies are in line with findings from a recent systematic review on the role of traumatic events, personality, and psychopathology in TS patients^9^, which highlighted how current literature could not lead to definitive conclusions on the role of psychological factors in TS. Specifically, Galli and colleagues suggested a role of long-lasting emotional distress in the contribution of TS exacerbation rather than contingent traumatic events preceding the onset of this clinical condition.

This hypothesis seems to be supported also by results from the current work. Whereas studies failed at showing associations between specific psychological features and HRV alterations, results indicated a possible role for two structured, long-lasting constructs, namely coping strategies and emotional arousal. A fundamental distinction must then be drawn between state and trait psychological characteristics. States are characteristic patterns of thinking, feeling, and behaving in a concrete situation at a specific moment in time, whereas traits are characteristic patterns of thinking, feeling, and behaving that generalize across similar situations, differ systematically between individuals, and remain rather stable across time^56^. Accordingly, results from the present review suggested that HRV alterations in TS may be mostly associated to trait, stable psychological dimensions (e.g., coping strategies and emotional arousal) rather than state, situational characteristics, although further investigations are warranted to better address this issue. Given their intrinsic characteristics, it is possible to hypothesize that dysfunctional traits observed after the acute event may be present also before TS exacerbation, may be associated to a psychophysiological imbalance in terms of HRV and thus may contribute as vulnerability factors for TS, as observed in other cardiovascular diseases^57–59^. In other words, in addition to answering which triggering, situational factors elicit stress response of the heart as suggested by the recent Expert Consensus Document on TS^6^, the relevant question appears to be which psychological traits, or *vulnerabilities,* may predispose to TS elapse and how they may be reflected and detected by autonomic assessments.

Results from the present work allow to draw some methodological considerations. In advancing the understanding and interpretation of HRV, it is imperative to address critical caveats for future investigations, particularly concerning associations between HRV and psychological profiles or markers in TS. Addressing these aspects could significantly improve the robustness and reliability of findings in this area.

First, in light of the findings and gaps identified within the existing literature, we propose several key recommendations aimed at optimizing methodologies and refining study designs to strengthen the quality and depth of future research in this domain. As reported in the quality assessment of the included articles, a major issue concerns the representativeness of the samples included in the studies: when samples are not representative of the general population, potential biases may emerge, affecting the external validity of the research and making it challenging to extrapolate the results to real-world scenarios or to make broad claims about the target population. Using random sampling techniques whenever possible, ensuring adequate sample sizes, and being transparent about the demographics and characteristics of the sample population may be some strategies to mitigate the impact of unrepresentative samples and improve the applicability of study findings to larger populations^60^. Also, the appropriate selection and careful definition of controls within scientific studies play a crucial role in ensuring the accuracy and reliability of research findings; defining controls accurately involves outlining clear criteria or characteristics that differentiate the control group from the experimental group, in accordance with the rationale guiding the study; moreover, it has been suggested that, in case–control studies, controls should not have history of the disease or condition of interest, and that a match for general characteristics recognized as influencing variables of interest, including sex and age, would be desirable^61,62^.

Secondly, the multifaceted nature of confounding variables that significantly influence HRV must be carefully considered and controlled for in future studies. Variables such as cardiac medication usage, cardiovascular fitness levels (measurable through weekly cardio training), overall health status, sex-specific considerations (including menstrual cycle phases), circadian influences, age, and other pertinent factors outlined in prior research^63^ require meticulous scrutiny. Regarding the articles included in the current work, these aspects have not always been fully addressed. For instance, age was not controlled for in 3 of the included studies^43,44,50^, whereas sex was not controlled for in 4 articles^42–44,50^; similarly, cardiac medication was not reported in 2 articles^44,50^ and information about overall health status was not included in 1 study^47^; notably, none of the included articles took into consideration fitness levels and circadian influences. Understanding the stability of these variables across time or their susceptibility to situational and individual variations, as well as their impact on patients’ autonomic and clinical outcomes is crucial, and future studies are invited to further address these issues.

Moreover, it is crucial to recognize and account for the impact of respiratory parameters on HRV metrics, including respiratory sinus arrhythmia (RSA), LF, and LF/HF. It has been observed that breathing, in terms of both tidal volume and duration of breaths, may affect HRV parameters independently from vagal activity^64–66^. In addition, it should be considered that respiratory parameters change as a function of psychological states^67^. Therefore, the use of established methodologies^68^ to evaluate and regulate these effects is essential. Nevertheless, the validity of RSA as an indicator of between-individual differences in vagal activation, as questioned by various studies^69,70^, warrants further exploration. Studies suggesting that baseline resting heart rate is a more reliable predictor of such differences^71^ advocate for the inclusion of heart rate findings alongside HRV outcomes. Consequently, it is recommended that both HRV metrics and corresponding heart rate results should be reported consistently, as proposed by previous research^69,72^, to ensure a more comprehensive understanding of autonomic nervous system activity in relation to psychological aspects in TS. These strategic considerations and methodological adjustments will substantially strengthen the validity and applicability of HRV investigations in elucidating the complex interactions between physiological and psychological factors in TS.

The main limitation of the current work is the limited number of studies included. The aim of the current systematic review and meta-analysis required specific inclusion criteria, which although reduced the number of available studies, ensured the formulation of a specific research question and a clear-cut synthesis of the topic. Moreover, the rigor of methodology we relied on (i.e., PRISMA guidelines) allowed to produce reliable results and conclusions. The issue concerning the limited number of retrieved articles and the small size of single case series is an objective limitation related to the different designs adopted by the included studies, which showed a median quality score of 5.5, pointing to the need for a better representativeness of the cases and selection/definition of controls in future studies. Nevertheless, to overcome this issue, the present study combined the qualitative, descriptive approach of a systematic review with the quantitative approach of a meta-analysis. More specifically, in the meta-analytic procedure, we adopted a multi-level meta-analysis approach, which allows to take into consideration multiple effect sizes within a single study, for a total of 41 ESs; moreover, despite the heterogeneity of ESs, The 2-level model showed an adequate fit to data (*χ*_(1)_ = 0.96; ns) and a null effect of moderators was found.

Limitations notwithstanding, this is the first study providing a systematic review and meta-analysis on the association between TS and autonomic alterations in the HRV domains. Furthermore, we also systematically assessed for the first time all available data on a possible relationship between HRV abnormalities and psychological factors in TS patients. Future research is warranted to further address the specific contribution of single HRV indices on TS elapse and prognosis, as well as more studies exploring the role of psychological vulnerabilities, rather than single traumatic stressors, in the association between HRV and TS.

## Method

The selection process of eligible articles to be included in the current work followed three steps: 1) a predefined algorithm was employed to search for suitable publications in electronic scientific databases; 2) after duplicates removal, a first screening was performed on titles and abstracts; survived articles were further considered according to predefined inclusion and exclusion criteria; 3) data of interest were extracted and analysed.

**Figure 1:**
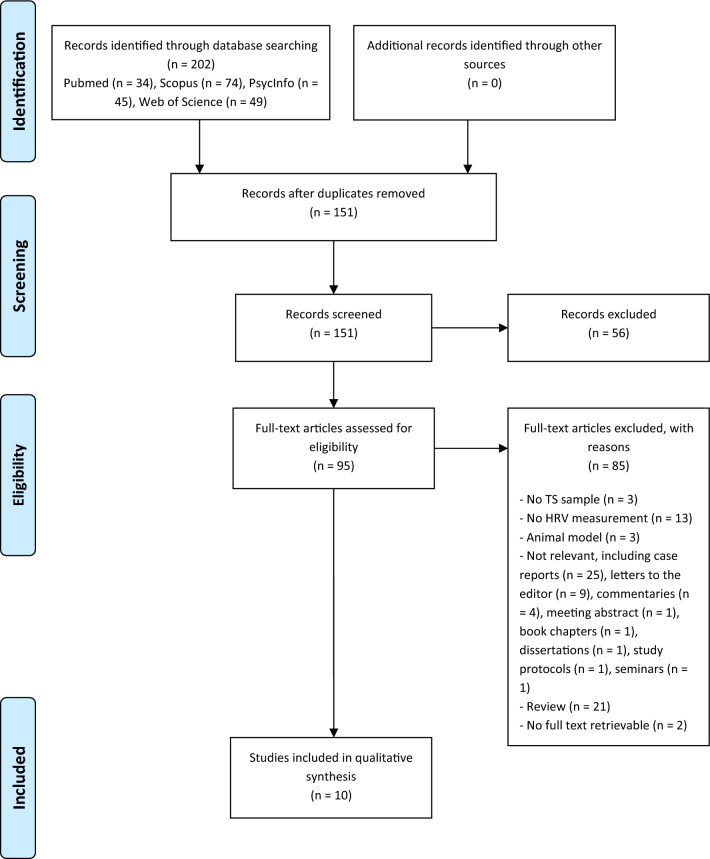
Detailed process of study selection. The diagram shows the detailed process of study selection in compliance with the PRISMA guidelines for systematic reviews and meta-analyses.

The present work was conducted in accordance with the PRISMA guidelines for systematic reviews and meta-analyses^73^ and the selection process was thus implemented in compliance with the PRISMA flow diagram (Fig. 1).

### Database search strategy

Electronic database search was conducted on the major databases in the field of health and social sciences: Pubmed (https://pubmed.ncbi.nlm.nih.gov/), Scopus (https://www.scopus.com/search/), PsycInfo (http://www.apa.org/pubs/databases/psycinfo/), and Web of Science (https://apps.webofknowledge.com). Databases were searched for scientific publication up until September 26, 2022, according to the following search algorithm: ((takotsubo) OR (tako-tsubo syndrome) OR (stress-induced cardiomyopathy) OR (takotsubo cardiomyopathy) OR (transient left ventricular ballooning syndrome) OR (apical ballooning syndrome) OR (ampulla cardiomyopathy) OR (broken heart syndrome)) AND ((hrv) OR (heart rate variability) OR (vagal tone) OR (vagal activity)). The search was limited to English-language publications.

### Literature search strategy and study eligibility

Once articles were retrieved according to the search algorithm, all duplicates were removed. Title and abstract of the survived publications were checked to exclude not relevant articles. Afterward, remaining articles were then screened according to predefined inclusion and exclusion criteria. Inclusion criteria were: a) studies with an analytical study design as defined by Grimes and Schulz^74^ (i.e., an observational study with a comparison or control group) or providing comparisons with normative data; both retrospective and perspective studies have been included to take into consideration the highest number of studies; b) diagnosis of TS according to the Mayo Clinic criteria or by the new TS criteria^75,76^; c) evaluation of one or more HRV indices either in the time-domain (e.g., SDNN, pNN50, RMSSD), in the frequency-domain (e.g., LF, HF, LF/HF), and/or non-linear analysis (e.g., SamEn, FD). Exclusion criteria were: a) case reports, reviews, letters to the editor, commentaries, meeting abstract, book chapters, dissertations, study protocols, seminars; b) studies written in other languages than English; c) surgical protocols or validation of measurement instruments; d) animal models; e) for pharmacological and behavioral intervention trials, only pre-intervention baseline measures were considered. When the full text was not retrievable, the article was excluded. After the application of inclusion and exclusion criteria, reference lists of survived articles were screened to search for additional relevant literature: in case of relevant citations, publications underwent the study eligibility process. The whole selection process was performed by two independent reviewers; in case of disagreement, reviewers discussed their view until a consensus was reached.

### Variables of interest and data extraction

Publications that survived the selection process were then analysed according to: a) authors and publication year, b) TS sample characteristics, c) control sample characteristics, d) time of assessment, e) HRV outcomes, f) psychological outcomes, g) study design, h) significant findings, i) quality assessment.

### Study quality assessment

The quality of the selected articles was evaluated using a quality index derived from the Newcastle–Ottawa Scale (NOS) for case–control studies on a 9-star model^77^. Relevant information and data from identified articles were independently extracted by two reviewers and used to calculate the quality index of each study.

### Meta-analytic procedures

The current meta-analysis was based on the Cohen’s *d* coefficient as an effect size measure. Values of d greater than or equal to 0.20, 0.50, and 0.80 were interpreted as small, moderate, and large effect sizes (ESs), respectively^78^. The index was primarily calculated using descriptive statistics reported in the Results section of each study. Meta-analytic procedures were based on the absolute value of ESs, which reflect the extent of HRV differences between patients with TS and HCs. Absolute values were chosen due to the fact that alterations of HRV captured by time- and frequency domains together with non-linear approaches are reflected by both heightened and reduced levels of each specific index. According to multiple HRV indexes estimated within each study included in the current work, we conducted a 3-level meta-analysis using the *metafor R* package^79^. The computation of model parameters was based on the restricted maximum-likelihood method^80^. The pooled ES (*d*_*w*_) was estimated assuming that ESs of each comparison (level 2) were nested within each study (level 3). Heterogeneity in effect sizes was computed through *Q* statistic^81^ and a multilevel version of *I*^2^ index^82^. The advantage of conducting a 3-level model was statistically demonstrated by comparing the Akaike (AIC) and Bayesian Information Criterion (BIC) indexes of a 3-level model with a reduced 2-level model together with the application of a Likelihood Ratio Test (LRT) between models. In presence of significant heterogeneity of effect sizes, 3-level mixed-effect models were estimated to test the impact of specific HRV indexes on the extent of pooled ESs. Furthermore, the 3-level meta-regression was conducted in order to evaluate confounding effects of other variables on ESs (i.e., year of publication, sample size, age, gender, length of HRV record, time of HRV assessment after the acute episode). Egger’s regression^83^ was estimated to detect publication biases. Bootstrap methodology (i.e., bias corrected and accelerated)^84^ was applied in computing the significance of the previous parameters.

### Supplementary Information

Supplementary Tables.

## Data Availability

The data that support the findings of this study are available from the authors upon reasonable request from the corresponding author.
