# Supplementary material for: Heart rate variability alterations in takotsubo syndrome and related association with psychological factors: a systematic review and meta-analysis
Source: Sci Rep. 2023 Nov 25;13:20744. doi: 10.1038/s41598-023-47982-0 (PMC10676391; doi:10.1038/s41598-023-47982-0)
Supplement: Supplementary file 1 — Supplementary Tables. [file 41598_2023_47982_MOESM1_ESM.pdf]

Supplementary Information

Heart rate variability alterations in Takotsubo syndrome and related association with psychological factors: a systematic review and meta-analysis

Gianluca Cruciani[a], Marco Cavicchioli[b], Gaetano Tanzilli[c], Annalisa Tanzilli[a,*], Vittorio Lingiardi[a,§,], Federica Galli[a,§]

Table S1

Synthesis of the main HRV parameters for the time domain, frequency domain, and non-linear indices

	Parameter	Unit	Description	Function
Time domain	Mean RR interval	ms	Time elapsed between two successive R-waves of the QRS signal on the electrocardiogram	Function of intrinsic properties of the sinus node as well as autonomic influences, with lower values indicating higher cardiac frequency
	SDNN	ms	Standard deviation of all NN intervals	It indicates the overall variability of the autonomic nervous system interplay, with lower SDNN pointing to lesser variability and thus little control of the parasympathetic over sympathetic system
	SDANN	ms	Standard deviation of the average normal-to-normal intervals for each of the 5 min segments during a 24 h recording	Lower values of SDANN indicate decreased parasympathetic activity
	SDNN index (SDNNi)	ms	Mean of the standard deviation of all the normal-to-normal intervals for each 5 minutes segment of a 24-h HRV recording	It primarily reflects autonomic influence on HRV
	RMSSD	ms	Root mean square of successive beat-to-beat interval differences	It reflects vagal regulation of heart rate, with higher RMSSD values indicating robust parasympathetic control over the sympathetic system
	pNN50	%	Percentage of successive intervals that differ by more than 50 ms	It reflects parasympathetic modulation of heart rate, with higher values indicating increased parasympathetic activity
	Triangular index (TI)		Geometric measure based on 24 h recordings which calculates	High values for TI suggest and enhanced vagally-mediated parasympathetic activity

			the integral of the density of the RR interval histogram divided by its height	
	TINN	ms	Triangular interpolation of the NN interval histogram	It represents the baseline width of a histogram displaying NN intervals
Frequency domain	VLF	ms^2	Very-low frequency band (between 0.0033 and 0.04 Hz)	It reflects circadian oscillations, body temperature, metabolism and the renin-angiotensin system, with increased VLF reflecting enhanced sympathetic activity
	LF	ms^2	Low frequency band (between 0.04 and 0.15 Hz)	It reflects a mix of sympathetic and vagally-mediated parasympathetic activity as well as baroreceptor activity while at rest, with conditions associated with sympathetic activation producing a decrease in the LF component
	HF	ms^2	High frequency band (between 0.15 and 0.40 Hz)	It reflects vagally-mediated parasympathetic activity and heart rate variations due to the respiratory cycle, with enhanced HF indicating robust parasympathetic control over the sympathetic system
	LF/HF	%	Ratio of LF to HF	It is an index that takes into consideration the fact that during 24-h HRV recording both the parasympathetic and sympathetic systems contribute to LF power and parasympathetic activity primarily contributes to HF power, with lower LF/HF ratios reflecting parasympathetic dominance.
Non-linear indices	DFA		Detrended Fluctuation Analysis	It extracts the correlations between successive RR intervals over different time scales, resulting in slope α1, which describes brief fluctuations and reflect the baroreceptor reflex, and slope α2, which describes long-term fluctuations and reflect regulatory mechanisms that limit fluctuation of the beat cycle: a decrease in each of the DFA's component is thought to represent a degree of the impaired cardiac response to external disturbances
	ApEn		Approximate entropy	It measures the regularity of a time series, with high ApEn values indicating low predictability of fluctuations and low ApEn indicating that the signal is regular and predictable
	SamEn		Sample entropy	It is a less biased measure of signal regularity, with lower values indicating its predactibility
	FD		Fractal dimension	It reflects the regularity of a signal and it has been linked to vagal modulation of heart rate
	1/f PLS		1/frequency power law slope	It is an index derived by the HRV power spectrum, and it has been associated with sympathetic modulation
	PRSA		Phase-rectified signal averaging	It allows an approximate distinction of sympathetic and vagal effects by separate assessment of deceleration-related (DC) and acceleration-related capacity (AC) of the heart rate time series

Table S2

Adapted version of the Newcastle-Ottawa scale used in the present study for quality assessment (maximum 9 stars).

**Selection (maximum 4 stars):**
Is the case definition adeguate?<br>Yes, according to the Mayo Clinic criteria or by the new TS criteria. *<br>No description<br>***Representativeness of the cases:***<br>Consecutive or obviously representative series of cases.*<br>Potential for selection biases or not stated.<br>***Selection of controls:***<br>Presence of one or more control groups. *<br>Absence of controls<br>***Definition of controls:***<br>No history of disease (healthy controls). *<br>Other diagnoses.
**Comparability (maximum 2 stars):**
Comparability of cases and controls on the basis of the design or analysis<br>Study controls matched for age and sex. **<br>Matching only for age or sex *<br>No matching or absence of controls
**Methods and procedure (maximum 3 stars):**
Experimental procedure:<br>The experimental procedure is well described and includes valid measures and methods. *<br>Poor description or no description of the procedure.<br>***Same experimental procedure for cases and controls:***<br>Yes. *<br>No or absence of controls<br>***Drop-outs rate:***<br>No drop-outs or same rate for all groups. *<br>Different rate or not defined or absence of controls

Table S3

Details on quality assessment indices for the retrieved studies

Articles	Selection				Comparability	Outcome (HRV and psychological tests)			
	**Adequate case definition**	**Representativeness**	**Selection of controls**	**Definition of controls**	**Comparability of cases and controls on the basis of the design or analysis**	**Experimental procedure description**	**Same experimental procedure for cases and controls**	**Drop-outs rate**	**Total NOS score**
Akashi et al., 2007	*	*	*	*	*	*	*	*	9
Bonnemeier et al., 2010	*	*	*	-	**	*	*	-	7
Collste et al., 2014	*	-	*	*	**	*	*	-	7
Krstacic et al., 2012	*	-	*	*	**	*	*	*	8
Lazzeroni et al., 2022	*	-	*	*	**	*	*	*	8
Mayer et al., 2016	*	-	-	-	-	*	-	-	2
Norcliffe-Kaufmann et al., 2016	*	-	*	*	*	*	*	*	7
Ortak et al., 2009	*	*	-	-	-	*	-	-	3

Waldenborg et al., 2011	*	*	-	-	-	*	-	-	3
Watson et al., 2022	*	-	*	-	**	*	*	*	7

Table S4

Meta-analytic results of psychophysiological indices alterations associated to TS.

Level 2 *N* of effect sizes	Level 3 *N* of studies	Moderators	*F* (df_1, df_2)	d_w (95% CI)	*Q* (*df*)	τ^2 Level 2<br>I^2 Level 2	τ^2 Level 3<br>I^2 Level 3	AIC	BIC	$\chi^2_{(1)}$	Egger's coefficient 95% Bootstrap CI
				**Absolute value of psychophysiological alteration**							
41	5			.91*** (.65 – 1.29)	238.28*** (40)	.81<br>85.36%	.003<br>.33%	121.83	126.89	.96	
41	-			.97*** (.66 - 1.29)		.82<br>85.67%		119.83	123.20		.73<br>(-4.68 – 2.83)
				**Psychophysiological indices absolute values**							
		1/f PLS		.92 (-.61 – 2.44)							
		AC		1.03 (-1.01 - 3.06)							
		ApEn		1.35 (-.39 - 3.09)							
		SamEn		.00 (-2.10 - 2.10)							
		DC		1.02 (-1.02 - 3.06)							
		DFA α1		3.74*** (1.07 - 5.77)							
		DFA α2		1.61 (-.35 - 3.57)							
		FD		1.45 (-.69 - 3.58)							
		H		3.95*** (1.89 - 6.00)							
41	-	HF	2.54*<br>(19, 21)	.56 (-1.05 - 2.17)	70.08***<br>(21)	.39<br>74.0%	-	92.58	114.51		
		LF		1.86* (.22 - 3.50)							
		LF/HF		.60 (-1.00 - 2.20)							
		TF		1.42 (-.72 - 3.54)							
		VLF		.22 (-1.87 - 2.32)							
		Mean NN		.00 (-2.08 - 2.08)							
		Mean RR		.68 (-1.04 - 2.40)							
		pNN50		.48 (-1.20 - 2.16)							
		RMSSD		.46 (-1.22 – 2.14)							
		SDANN		.54 (-1.06 - 2.14)							
		SDNN		.67 (-.94 - 2.28)							
